# Formal analysis of imprecise system requirements with Event-B

**DOI:** 10.1186/s40064-016-2657-8

**Published:** 2016-07-07

**Authors:** Hong Anh Le, Shin Nakajima, Ninh Thuan Truong

**Affiliations:** Hanoi University of Mining and Geology, Bac Tu Liem, Hanoi, Vietnam; National Institutue of Informatics, 2-1-2 Hitotsubashi, Chiyoda-ku, Tokyo, Japan; VNU, University of Engineering and Technology, Cau Giay, Hanoi, Vietnam

**Keywords:** Imprecise requirements, Event-B, Analysis

## Abstract

Formal analysis of functional properties of system requirements needs precise descriptions. However, the stakeholders sometimes describe the system with ambiguous, vague or fuzzy terms, hence formal frameworks for modeling and verifying such requirements are desirable. The Fuzzy If–Then rules have been used for imprecise requirements representation, but verifying their functional properties still needs new methods. In this paper, we propose a refinement-based modeling approach for specification and verification of such requirements. First, we introduce a representation of imprecise requirements in the set theory. Then we make use of Event-B refinement providing a set of translation rules from Fuzzy If–Then rules to Event-B notations. After that, we show how to verify both safety and eventuality properties with RODIN/Event-B. Finally, we illustrate the proposed method on the example of Crane Controller.

## Introduction

Requirement engineering is a process of specifying, analyzing, and checking provided services and constraints of a system. It is one of the most significant steps in software development. System requirements aim to take into account various demands of all the stakeholders, where detecting and resolving conflicts is important. The requirements sometimes include imprecise descriptions where ambiguous, vague or fuzzy terms, such as “very good”, “far”, or “less important”, are used. This is because the stakeholders do not care much about describing the system precisely or imprecise requirements are more suitable in some contexts. In software development, imprecision in the requirement specification also causes many problems. Formal specification methodologies, however, require the requirements to be described precisely. Hence, there is a gap between imprecise requirements and formal specification methods. Therefore, frameworks which are formal enough to be used for analyzing as well as representing imprecise requirements are desirable.

The method with Fuzzy sets, proposed by Zadeh (1965), is one such formal framework, where the Fuzzy If–Then rules are sometimes employed to represent imprecise system requirements. Informal statements expressed in natural languages such as “very far” or “too close” can be naturally captured using Fuzzy sets, which enables further analysis on the specifications. The analysis involves continuous numerical reasoning since the Fuzzy set is essentially based on the idea of representing the fuzziness degree in terms of Real numbers between 0 and 1.

In general, system requirements include functional specifications, whose various properties are checked at this same level of abstraction before starting further development steps. The requirements written in terms of Fuzzy If–Then rules can be an adequate representation, but require further techniques for checking properties formally, which may elucidate perspectives different from those for detecting and resolving conflicts of the requirements. The Fuzzy If–Then rules have been translated into other formal frameworks such as PetriNet (Intrigila et al. 2005; Yang et al. 2003) or Z notation (Chris and Paul 2003). Unfortunately, these existing approaches have disadvantage in that they do not provide adequate verification methods for temporal properties such as safety or eventuality. The existing approaches are discussed in more detail in “Related work” section.

This paper employs Event-B and its refinement-based modeling approach for specification and verification of both safety and eventuality properties when the requirements are represented by the Fuzzy If–Then rules. In particular, we apply the proof methods proposed in Hoang and Abrial (2011) to verify the eventuality properties. Our prior work (Le et al. 2014) initially proposed to use Event-B to formalize imprecise requirement. It provided the basic result of checking safety property of imprecise requirement using Event-B. This paper reports the concrete results of formal checking of both safety and eventuality properties for imprecise system requirements. The contributions of the paper are as follows: (1) providing a presentation of fuzzy terms in classical set theory, (2) providing a set of translation rules from Fuzzy If–Then rules to Event-B language constructs, which makes use of the refinement modeling approach that Event-B supports, and (3) demonstrating how both safety and eventuality properties of a set of the Fuzzy If–Then rules can be verified using RODIN (Abrial et al. 2010), a supporting tool for Event-B.

The rest of the paper is structured as follows. Section “Backgrounds” provides some background of fuzzy sets, fuzzy If–Then rules, and Event-B. In “Imprecise requirements analysis with Event-B” section, we give a representation of fuzzy sets in classical sets. Using such representation, we first introduce a set of translation rules to model fuzzy If–Then rules by Event-B. Then, we propose a refinement-based modeling method to specify and check eventuality properties. In fourth section presents the example of a crane controller to illustrate the proposed method in detail. We summarize “Related work” in fifth section. “Conclusions” are given in final section.

## Backgrounds

In this section, we briefly introduce an overview of fuzzy logics (in the broad sense) that mainly serve for describing and analyzing impreciseness. We also summarize basic knowledge of Event-B.

### Fuzzy sets and fuzzy If–Then rules

In order to deal with systems which are too complex or too ill-defined to admit of precise descriptions, Zadeh (1965) introduces a logical framework which is not traditional two-valued, but multi-valued logics whose values are interpreted by Fuzzy sets.

Fuzzy sets are actually functions that map a value that might be a member of a set to a number between zero and one indicating its actual degree of membership. A fuzzy set F defined on a universal set X is a set, each element of which is a pair of values $(x,\mu _F(x))$, where $x \in X$ and $\mu _F(x): X \rightarrow [0,1]$.

Fuzzy sets use so-called linguistic variables in addition to numerical variables. The values of a linguistic variable are labels of fuzzy subsets of *X* which have the form of phrases or sentences in a natural or artificial language. For example, *height* is a linguistic variable labeled x, and the values of x might be “tall”, “not tall”, “very tall”, or “tall but not very tall”. Generally, a value of a linguistic variable is a concatenation of atomic terms that can be divided into main categories shown below:

- Primary terms: which are labels of specified fuzzy subsets of the universal set (for instance: *tall* in the above example).
- Hedges: such as “very”, “slightly”, etc.
- Negation and connectives symbols (i.e *not*, *and*, *or*).

A fuzzy hedge is an operator which transforms the fuzzy set *F*(*x*) into the fuzzy set *F*(*hx*). The hedges are the functions that generate a larger set of values for linguistic variables. For instance, using hedge *very* along with negation *not* applied to the term *tall*, we can have *very tall* or *not very tall*.

A more general concept, which plays an important role in the fuzzy sets approach to analyzing imprecise description, is Fuzzy If–Then rules. They are mainly used for specifying behavior of the system. It has a form: IF x is A THEN y is B where *A* and *B* are fuzzy sets; *x* and *y* are linguistic variables. Here is an example: IF the weather is bad THEN the speed is slow.

### Event-B and Rodin

#### Event-B

Event-B Abrial (2010) is a formal method for system-level modeling and analysis. Key features of Event-B are the use of set theory as a modeling notation, the use of refinement to represent systems at different abstraction levels and the use of mathematical proofs to verify consistency between refinement levels. A basic structure of an Event-B model consists of MACHINE and CONTEXT.

An Event B CONTEXT describes a static part where all the relevant properties and hypotheses are defined. A CONTEXT consists of carrier sets, constants, axioms. Carrier sets, denoted by *s*, are represented by their names, and are non-empty. Different carrier sets are completely independent. The constants *c* are defined by means of a number of axioms *P*(*s*, *c*) also depending on the carrier sets *s*.

A MACHINE is defined by a set of clauses. A machine is composed of variables, invariants, theorems and events. Variables *v* are representing states of the model. Invariants *I*(*v*) yield the laws that state variables *v* must always satisfy. These laws are formalized by means of predicates expressed within the language of First Order Predicate Calculus with Equality extended by Set Theory. Events *E*(*v*) present transitions between states. Each event has the form $evt =$**any***x***where***G*(*x*, *v*) **then**$A(x,v,v')$**end**, where *x* are local variables of the event, *G*(*x*, *v*) is a guard condition and $A(x,v,v')$ is an action. An event is enabled when its guard condition is satisfied. The event action consists of one or more assignments. We have three kinds of assignments for expressing the actions associated with an event: (1) a deterministic multiple assignment ($v:= E(t,v)$), (2) an empty assignment (skip), or (3) a non-deterministic multiple assignment ($v :| P(t,v,x')$).

To deal with complexity in modeling systems, Event-B provides a refinement mechanism that allows us to build the system gradually by adding more details to get a more precise model. A concrete Event-B machine can refine at most one abstract machine. A refined machine usually has more variables than its abstraction as we have new variables to represent more details of the model. In superposition refinement, the abstract variables are retained in the concrete machine, with possibly some additional variables. In data refinement, the abstract variables *v* are replaced by concrete ones *w*. Subsequently, the connections between them are represented by the relationship between *v* and *w*, i.e. gluing invariants *J*(*v*, *w*).

In order to check if a machine satisfies a collection of specified properties, Event-B defines proof obligations (POs) which we must discharge. Some of the proof obligations relevant to discussion here are invariant preservation (INV), convergence (VAR), deadlock-freeness (DLF). INV PO means that we must prove that invariants hold after event’s execution. The proof obligation is as follows: $I(v),G(x,v),A(x,v,v') \vdash I(v')$, where $v'$ is value of variable *v* after executing the event. VAR PO means that events cannot take control forever. To prove this, we use a variant *V* which is mapped to a finite set, then this variant is proved to be decreased in each convergent event. It is described as follows.

$I(v),G(x,v),A(x,v,v') \vdash V(v') \subset V(v)$. Deadlock-freeness for a machine ensures that there are always some enabled events during its execution. Assume that a machine contains a set of n events $e_i ( 1 \le i \le n)$ of the following form: $evt =$ any *x* where *G*(*x*, *v*) then $A(x,v,v')$ end. The proof obligation rule for deadlock-freeness is as follows: $I(v) \vdash \bigvee \nolimits _{i=1}^n (\exists x_i \cdot G(x_i,v))$.

Event-B provides ways to express safety properties directly by using the invariants. While safety properties guarantee that bad things do not happen, an eventuality property is one of liveness properties assuring that the system will reach a defined good state. Event-B does not support to specify liveness properties directly but we can follow the approach (Hoang and Abrial 2011) to verify properties such as *existence* ($\square \lozenge P$), *progress* ($\square (P_1 \implies \lozenge P_2)$), or *persistence* ($\lozenge \square P$), where *P* is any first order logic formula, $\lozenge$ and $\square$ are standard operators of Linear Temporal Logic (LTL), under weak-fairness assumption. We will discuss here in detail *existence* property. Assume that a given machine *M* with *n* events $e_i (1 \le i \le n)$, $e_i =$ any *x* where *G*(*x*, *v*) then $A(x,v,v')$ end. They claim that if *M* is convergent in $\lnot P$ and *M* is deadlock-free in $\lnot P$, then $\square \lozenge P$ is satisfied in *M*. This approach uses the variant clause to prove convergence of a machine and we introduce an auxiliary refined machine at the last refinement step to apply this proof method.

#### Rodin

**Fig. 1:**
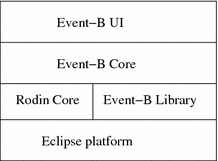
Rodin tool architecture

Rodin, an extension of the Eclipse platform, allows to create Event-B models with an editor. It also automatically generates the proof obligations of a model that can be discharged automatically or interactively. The architecture of the tool is illustrated in Fig. 1. Event-B UI provides users interfaces to edit Event-B models. Event-B Core has three components: static checker (checking the syntax of Event-B models), the proof obligation generator (producing simplified proof obligations that make them easier to discharge automatically), and the proof obligation manager (manging proof obligations and the associated proofs). The Rodin Core consists of two components: the Rodin repository (managing persistence of data elements) and the Rodin builder (scheduling jobs depending on changes made to files in the Rodin repository).

## Imprecise requirements analysis with Event-B

First, this section presents an approach to specifying imprecise requirements in classical set theory. This representation is the basis of its formalization in Event-B. After that, we introduce a new refinement-based approach to analyzing eventuality properties of imprecise system requirements.

### Presentation of imprecise requirements in classical sets

As stated above, fuzzy sets can be used as the foundation for representing imprecise requirements. The behavior of such requirements can be described by Fuzzy If–Then rules. We will show that imprecise requirements, which are described by Fuzzy If–Then rules, can be represented in classical sets.

First, the general form *FR*, also called well-defined form, of an imprecise requirement can be represented as:

$${\hbox {IF }} x {\hbox { is }} \delta Y_i {\hbox { THEN }} m \,{\hbox { is }} \gamma P_i$$

where *x* and *y* are linguistic variables, $Y_i \in Y$ and $P_i \in P$ are fuzzy values, and $\delta$ and $\gamma$ are fuzzy hedges which are applied on the fuzzy sets *Y* and *P* respectively.

#### **Definition 1**

(*Imprecise requirement*) An imprecise requirement is defined as a 6-tuple $FR=\langle x, m, \delta , \gamma , Y_i,P_i \rangle$, where *x* and *m* are linguistic variables, $\delta$ and $\gamma$ are fuzzy hedges, and $Y_i$ and $P_i$ are fuzzy values.

Recall that, in classical set theory, sets can be combined in a number of different ways to produce another set such as Union, Intersection, Difference, or Cartesian product. Below we recall some definitions related to Cartesian product of multiple sets is also defined using the concept of n-tuple.

#### **Definition 2**

(*ordered n-tuple*) An ordered n-tuple is a set of n objects with an order associated with them. If *n* objects are represented by $x_1$, $x_2, \ldots$, $x_n$, then we write the ordered n-tuple as $\langle x_1, x_2, \ldots , x_n \rangle$.

#### **Definition 3**

(*Cartesian product*) Let $A_1, \ldots , A_n$ be *n* sets. Then the set of all ordered n-tuples $\langle x_1, \ldots , x_n \rangle$ , where $x_i \in A_i$, $\forall i$, $1 \le i \le n$, is called the Cartesian product of $A_1, \ldots , A_n$, and is denoted by $A_1 \times \cdots \times A_n$.

#### **Proposition 1**

*A set of well-defined imprecise requirements can be specified by classical sets.*

#### *Proof*

Suppose that, imprecise requirements of a system are specified by $FR = \{FR_i\}$, $FR_i = \{x_i, m_i, \delta _i, \gamma _i, Y_i, P_i\}$, $1 \le i \le n$. Clearly that, $x_i,m_i$ are considered as elements of variables sets, $Y_i$ and $P_i$ belong to fuzzy sets. We consider if $\delta _i Y_i$ can be specified by a classical set in which $\delta _i$ is a hedge and $Y_i$ is a value in fuzzy set *Y*. As mentioned in “Fuzzy sets and fuzzy If–Then rules” section, $\delta _i$ transforms fuzzy set *Y* to another fuzzy set. Moreover, according to the Definition 3, $\delta _i Y_i$ is a membership of the Cartesian product of two sets $\delta \times Y$. Similarly with the case of specifying $\gamma _i P_i$. Consequently, every element in *FR* can be specified by classical sets. $\square$

### Modeling imprecise requirements

We will explain how imprecise requirements described by Fuzzy If–Then rules are modeled based on their new representation. Suppose that, a system is specified by a set of requirements ${\mathbf{FR}}_i$ :

$${\mathbf{if}} \,\,x_i \,{\hbox {is}}\, \delta _i Y_i\, {\mathbf{then}}\,m_i {\hbox { is }} \gamma _i P_i\;{\hbox{end}}$$

According to the Proposition 1, the above requirements can be represented by classical sets. Next, we take into account the semantic of Fuzzy If–Then rules. In fact, these rules can be interpreted in various ways. In this paper, we define the semantic of a rule as follows:

$$\forall x_i,y_i \circ (x_i = \delta _i \mapsto Y_i) \implies (y_i = \gamma _i \mapsto P_i)$$

where $x_i$ and $m_i$ are linguistic variables, $\gamma _i$ and $\delta _i$ are hedges, and $Y_i$ and $P_i$ are fuzzy sets. It is informally interpreted as if $x_i$ is equal to pair $\langle \delta _i,Y_i \rangle$, then $y_i$ is equal to pair $\langle \gamma _i,P_i\rangle$.

Since Event-B is a language based on the classical set theory, we propose an approach to modeling the system with Event-B method. A system consisting a collection of requirements $FR_i, 1 \le i \le n,$ is modeled by an Event-B model $FR_B=\langle FR\_C,FR\_M \rangle$, where $FR\_C$ and $FR\_M$ are Event-B context and machine respectively. We propose below translation rules to map imprecise requirements to Event-B’s elements. The important principle of the translation process is that we can preserve the structure and represent all imprecise requirements using the Event-B notation. Moreover, safety properties must be preserved by actions of the system.

*Translation rules*

- Rule 1: All hedges $\delta _i$ and $\gamma _i$, fuzzy values $Y_i$ and $P_i$ in the set of requirements are translated to three sets *H*, *Y*, and *P* respectively. They are stated in the SETS clause of $FR\_C$.
- Rule 2: Linguistic variables $x_i$ and $m_i$ in each $FR_i$ are mapped to variables $x_i$ and $m_i$ of the Event-B machine $FR\_M$.
- Rule 3: Each variable $x_i$ is described as a member of a Cartesian product of two sets $H \times Y$; $m_i$ is described as a member of a Cartesian product of two sets $H \times P$ (Proposition 1).
- Rule 4: Each requirement $FR_i$ is modeled by an event $ev_i$ in Event-B machine $FR\_M$. If-part of the requirement becomes guard of the event, then-part is translated to event’s action.
- Rule 5: Safety properties of the system are modeled as invariants ${\mathcal {I}}$ of the machine $FR\_M$.

*Translation correctness*

Let $FR_i = \{x_i, m_i, \delta _i, \gamma _i, Y_i, P_i\}$ be a Fuzzy If–Then rule. According to Rule 1, 2, 3 and 4, it is translated to an event **when**$x_i$ = $\delta _i \mapsto Y_i$**then**$m_i := \gamma _i \mapsto P_i$, i.e., $x_i = \delta _i \mapsto Y_i \implies m_i := \gamma _i \mapsto P_i$. As a consequence, the translation rules preserve the semantic of a Fuzzy If–Then rule.

**Fig. 2:**
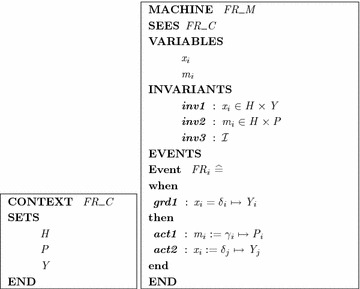
A part of Event-B specification for imprecise requirements modeling

Note that, these are only partial transformation rules, we need to give more additional parts to obtain the completed Event-B specification (Fig. 2).

#### **Proposition 2**

*With the modeling proposed in translation rules, the safety properties are preserved by all actions of imprecise system requirements.*

#### *Proof*

Suppose that, a collection of imprecise requirements $FR = \{FR_i\}$, $1 \le i \le n$, are translated to *n* corresponding events $evt_i$. Safety properties of the system are specified in the invariant ${\mathcal{I}}$. We have to prove that safety constraints are preserved through all requirements by showing that it remains true before and after firing (executing) each event. This is obviously achieved through proof obligations of the Event-B machines which is used to preserve their invariants.

Without loss of generality, we assume that the imprecise requirements and constraints contain one variable *v*, hence we need to prove:

$${\mathcal {I}}(v) \wedge evt_i(v,v') \vdash {\mathcal {I}}(v')$$

This predicate allows us to ensure the safety properties after executing the events in model, which is exactly the form of INV proof obligation generated from Event-B machine. Therefore, the safety properties stated in requirements are shown preserved if the corresponding INV proof obligation is proved. $\square$

### Modeling eventuality properties with refinement

Hoang and Abrial (2011) introduced reasoning techniques to prove classes of liveness properties such as *existence*, *progress*, *persistence*. They claims that with a state formula *R* which is a first-order logic formula and an Event-B machine *M* that is convergent and deadlock-free in *R* then $\lnot R$ will *always eventually* ($\square \lozenge \lnot R$) hold.

In order to reason about eventuality properties on a set of fuzzy If–Then rules, we initially presented the method (Le et al. 2015) following the techniques introduced in Hoang and Abrial (2011). We first map fuzzy values to Natural numbers. Since fuzzy sets can be represented by classical sets consisting of discrete values (“Presentation of imprecise requirements in classical sets” section), the mapping on Natural numbers instead of a continuous range [0..1] is acceptable. Therefore, we give a new definition of fuzzy sets as follows

#### **Definition 4**

(*Fuzzy set*) A fuzzy set is a pair $\langle U,\mu \rangle$, where *U* is a set and $\mu$ is the membership degree function, can be represented as a pair $\langle P,\mu _s \rangle$, where *P* is a crisp set, $\mu _s$ is a total function such that $\mu _s: P \rightarrow {\mathbb {N}}$

Similarly, we also use a total function $\mu _H: H \rightarrow {\mathbb {N}}$ as mapping values of fuzzy hedges.

Recall that, a system is specified by a collection of requirements ${\mathbf{FR}}_i$:

$${{\mathbf{if}}}\, x_i {\hbox { is }} \delta _i Y_i\;{\mathbf{then}}\;m_i {\hbox { is }} \delta _j P_i$$

We propose a refinement-based approach to modeling with an introduction of additional translation rules to extend the context and to refine the machine of the abstract model as follows

- Rule 6: Fuzzy values of each element in *P*, *Y* and hedges $\delta$ are translated to total functions $deg_P: P \rightarrow {\mathbb {N}}$, $deg_Y: Y \rightarrow {\mathbb {N}}$, and $deg_H: H \rightarrow {\mathbb {N}}$ respectively.
- Rule 7: Adds a variant mapping to linguistic variable $x_i$ that appears in eventuality property expression $Q(x_i)$.
- Rule 8: Refines each event representing one Fuzzy If–Then rule by two events: a convergent and an ordinary one.
- Rule 9: Adds a clause $\lnot Q(x_i)$ to the guards of each convergent event, and a clause $Q(x_i)$ to the ordinary one.

**Fig. 3:**
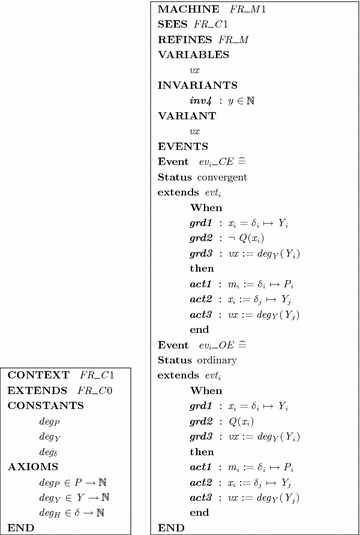
A part of Event-B specification for eventuality property modeling

A partial Event-B specification for these rules is depicted in Fig. 3.

Before showing that if a collection of requirements satisfy a eventuality property *Q*(*x*), we introduce definitions relating to some properties of fuzzy rules.

#### **Definition 5**

(*Convergence*) A set of fuzzy rules are convergent from a state *C*(*x*) if each rule decreases value of variable x. It is formally defined as: $FR_i,C(x) \vdash x' < x$, where $x'$ is value after executing rule $FR_i$.

#### **Definition 6**

(*Deadlock-freeness*) A set of fuzzy rules are deadlock-free in a state *R*(*x*) if IF clause of at least one rule is satisfied. It is formally defined as $R(x) \implies \bigvee \nolimits _{i=1}^n (\exists x_i.x_i = \delta Y_i).$

#### **Proposition 3**

*If a collection of Fuzzy If–Then rules*$\{FR\}$*are convergent and deadlock-free from a first-order logic state formulaR*(*x*), *wherexis a linguistic variable, then the state property*$\lnot R(x)$*will always eventually holds. Formally, we have*$\{FR\} \vdash \square \lozenge \lnot R(x)$.

#### *Proof*

Suppose that, a set of fuzzy If–Then rules $FR = \{FR_i\}$, $1 \le i \le n$, is first formalized by an abstract machine $M\_0$, then is refined by another machine $M\_1$ containing a set of convergent events $evt_i$.

Applying Rule 8, each fuzzy rule is represented by a convergent event $evt_i$ with guard *G*(*x*). Following Rule 9, a new clause $\lnot R(x)$ is added to the guard condition of each convergent event.

According to the translation Rule 6 and 7, approximation of a linguistic variable *x* is a natural number and is mapped to an variant *V*(*x*). Furthermore, each fuzzy rule decreases the fuzzy variable *x* (Definition 5), i.e $V(x') < V(x)$. Hence, we have

$${\mathcal {I}},G(x),\lnot R(x) \vdash V(x') < V(x).$$

This predicate is the form of VAR proof obligation generated from Event-B machine to prove that all events of the machine $M\_1$ are convergent (*).

We already state that fuzzy rules are deadlock free in *R*(*x*), according to Rule 8, Rule 9 and Definition 6 we have: $R(x) \implies \bigvee \limits _{i=1}^n (\exists i\cdot G(ev_i))$. This predicate is the form of DLF proof obligation generated from Event-B machine to prove machine $M\_1$ is deadlock-free in *R*(*x*)(**).

From (*) and (**), based on the reasoning technique in Hoang and Abrial (2011), we have a conclusion: $\{FR\} \vdash \square \lozenge \lnot R(x)$. $\square$

## An example: Container Crane Control

In this section, first we introduce an example of Container Crane Control (Fuzzytech 2012), then follow the proposed method in “Imprecise requirements analysis with Event-B” section to model and verify the safety and eventualities properties.

### Example description

**Fig. 4:**
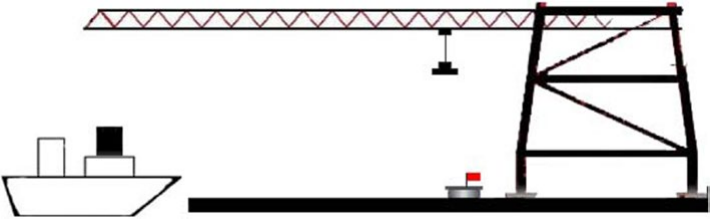
Container Crane Control system

Container cranes are used to load and unload containers on a ship in most harbors. They pick up single containers with cables that are mounted on the crane head (Fig. 4).

The crane head moves on a horizontal track from a starting position. The speed of the crane head is controlled by a motor power with a speed level. We start the motor with a fast speed. If the crane head is still far away from the container, we adjust the motor power to a medium speed. If the crane head is in a distance nearer to the target, we reduce the speed to slow. When the container is close to the target position, the speed should be very slow. When the container is above the container, we stop the motor. The crane head loads containers and goes back to the start position. The system has a safety property such that the speed of motor can not be high if the target is not far (property ${\mathcal {I}}$). The system needs to satisfy that the crane head eventually is above the container (property ${\mathcal {Q}}$).

From this description of the system, a collection of imprecise requirements *FR* is extracted as follows:

- $FR_1$: If the crane is at starting position, then power is fast level.
- $FR_2$: If the distance to the container is far, then power is medium level.
- $FR_3$: If the distance to container is medium, then power is adjusted to slow level.
- $FR_4$: If the distance is close, then power is very slow level.
- $FR_5$: If the crane is above the container, then power is stopped.

Then we have to check if $\{FR\} \vdash {\mathcal {I}}$ and $\{FR\} \vdash \square \lozenge {\mathcal {Q}}$.

### Modeling Container Crane Control system

#### Modeling the system with safety property

Applying the translation rules presented in “Modeling imprecise requirements” section, we first translate the set of requirements to the Event-B context as follows:

- Apply *Rule 1*: Fuzzy hedges and fuzzy values in the requirements are translated into the sets HEDGES, DISTANCE, and POWER of the Event-B context $Crane\_C0$.

Context $Crane\_C0$ is presented partially as follows 
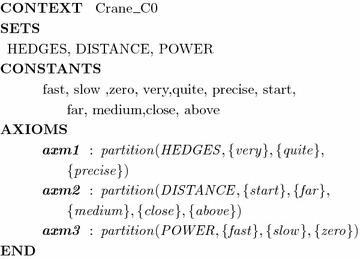


We continue to formalize the dynamic part of the model with the following translations.

- Apply *Rule 2*: Linguistic variables in the requirements are translated into Event-B constructs such as *distance* and *power*. According to Rule 3, types of these two variables are represented by invariants *inv*1 and *inv*2.
- Apply *Rule 4*: Each imprecise requirement $FR_i$ of the system is translated to an EVENT $evt_i$, $1 \le i \le 5$. More specifically, the imprecise requirement *r*4 is translated to $evt_4$ illustrated in the machine $Crane\_M0$. The other requirements are translated similarly. Moreover, in the initial states, *distance* is equal to *start* and *power* is stopped (modeled in Initialisation event).
- Safety property is stated as invariant *inv*3 (Rule 5).

The machine $Crane\_M0$ is described partially as follows: 
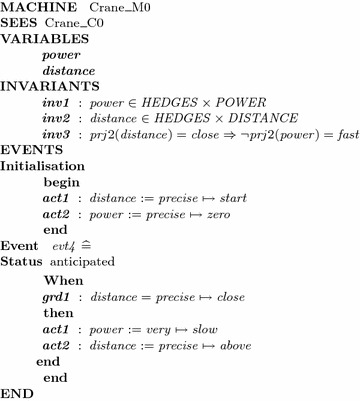


#### Refinement: modeling eventuality property

We refine the abstract model by following the method described in “Modeling eventuality properties with refinement” section to model the desired eventuality property. First, we apply Rule 6 to extend the abstract context Crane_C0 to define Crane_C1 by introducing three total functions for numerical values of fuzzy sets. The specification of this context is partially described as follows: 
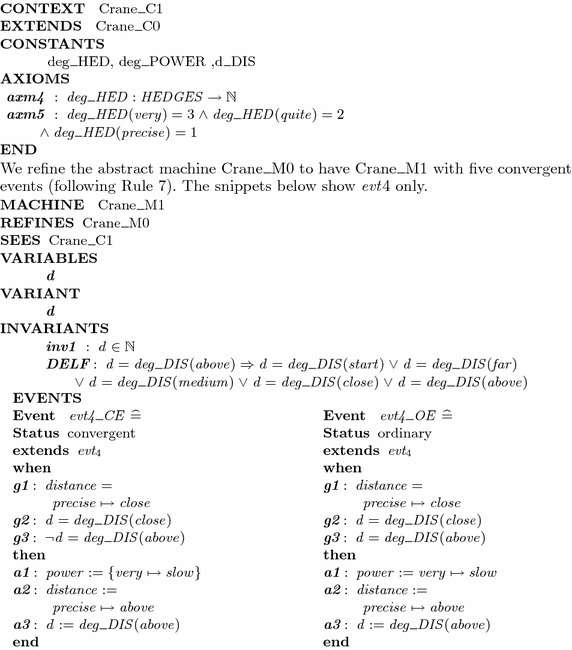


### Checking properties

The system has a safety property which is formalized as an invariant clause $inv3:prj2(distance)=close \implies \lnot prj2(power)=fast$. Its proof obligations are generated and discharged automatically using the Rodin tool under the label $evt_i/inv3/INV, 1 \le i \le 5$. It ensures that invariant is preserved through events, i.e., the requirements of this system conform to the safety property.

While safety property is maintained in every refinement, eventuality can only be verified in the machine $Crane\_M1$. Hence, we have to prove that $Crane\_M1 \vdash \square \lozenge (d=deg\_DIS(above))$. The deadlock-free property of this machine is encoded as the theorem *DELF* in $Crane\_M1$. Its proof obligation is generated as *DELF* / *THM*. In order to check the convergent property, proof obligations are generated for each convergent events of machine $Crane\_M1$ ($evt_i/NAT$ and $evt_i/VAR$). The abstract machine $Crane\_M0$ generates six *INV* proof obligations. The refined machine $Crane\_M1$ generates two proof obligations for dead-lock freeness and ten proof obligations for convergence property. All proof obligations are discharged automatically in the Rodin tool.^1^

## Related work

In this section, we classify the related papers into several categories. The first one consists of the research work making use of fuzzy set and fuzzy logic to analyze imprecise requirements. The second one consists of the results that use formal methods to model fuzzy sets and Fuzzy If–Then rules. The papers in third group handle with self-adaptive systems modeling.

In early of 90s, Liu and Yen (1996) proposed to use fuzzy sets and fuzzy logics as the foundation for analyzing imprecise requirements. They use fuzzy logic to resolve the conflicts between imprecise requirements. They treated imprecise requirements as a collection of fuzzy sets, i.e., the requirement has the form of “A is B” where *A* and *B* are fuzzy sets. Applying this result, a tool named STAR was developed for analyzing imprecise requirements (Yen et al. 1998).

In another research direction, formal methods have been used for specifying fuzzy terms. Matthews introduced a fuzzy logic toolkit for the formal specification language Z (Matthews and Swatman 2000). This toolkit defines the operators, measure and modifiers necessary for the manipulation of fuzzy sets and relations. A series of laws are provided that establish an isomorphism between conventional Z and the extended notation when applied to boolean sets and relation. It can be modeled as a partial rather than total function. The focus is on the specifications of the rule base and the operations necessary for fuzzy inferences. However, they do not incorporate the notion of refinements. It just provides definition and manipulation of fuzzy sets and relations by using Z.

Pavliska et al. (2006) introduced modified Petri Nets as a tool for fuzzy modeling. Basic concepts and relations between Fuzzy Petri Nets and Fuzzy If–Then rules are described and an algorithm for decomposition of fuzzy Petri net into set of linguistic descriptions are presented and its implementation mentioned. Their work just showed how to model the system and does not mention how to verify the system properties.

Intrigila et al. (2005) have introduced a verification method of fuzzy control systems using model-checking technique with Murphi verifier. The authors eased the modeling phase by using finite precision real numbers and external C functions.

Yang et al. (2003) proposed to use high-level Petri Net in order to verify fuzzy rule-based systems. This method can detect the system’s errors such as redundancy, inconsistency, incompleteness, and circularity but it has to take extra step to normalize the rules into Horn clauses before transforming these rules to and use incidence matrix as fixed-value matrix for degree membership.

When modeling uncertain behavior of the self-adaptive software systems, the vague, uncertain, and imprecise requirements also raise issues.

Whittle et al. (2009) proposed a new specification language, named RELAX, for self-adaptive systems. It is expressive language based on fuzzy branching temporal logic to specify the uncertain dynamic behavior of the system. The paper, however, neither shows the verification phase nor provide support tool.

Han et al. (2014) introduced an approach (FAME profile) to modeling fuzzy self-adaptive software systems by extending UML profile. The authors incorporate four kinds of new constructs into UML meta models. With the provided tool, the approach supports for modeling such systems well. In comparison with this paper, our approach aims at not only modeling the Fuzzy If–Then rules but also detecting conflicts. After the modeling process, safety and eventuality properties of the system can be verified. These points are not mentioned in Han et al. (2014).

## Conclusions

Although imprecise requirements are often found in software development processes, few work have been addressing the problem of modeling and verifying such descriptions so far. This paper presented a new specification and verification framework, in which the requirements were modeled in the Fuzzy If–Then rules. The rules were translated into a set of Event-B descriptions so that the refinement-based modeling method could be applied for the verification. With the proposed method, we can verify the safety and eventuality properties of the system described by imprecise requirements. We proposed to use classical set to represent Fuzzy If–Then rules and this representation is sufficient to analyze such properties in Event-B. We showed that the verification was mostly conducted automatically using the current RODIN tool. However, due to some limitation of the reasoning technique, we can only check the eventuality properties at the last refinement. One of the future work is to study a method for verifying eventuality properties at every refinement stage. Analyzing time dependent properties following the approach presented in Abrial et al. (2012) is also one of our future research direction.

## Footnotes

1 Rodin project archive: http://uet.vnu.edu.vn/~thuantn/CraneController.zip.
